# Impact of antibacterial detergent on used‐towel microbiomes at species‐level and its effect on malodor control

**DOI:** 10.1002/imt2.110

**Published:** 2023-06-05

**Authors:** TzeHau Lam, Yuxiang Liu, Fumi Iuchi, Yolanda Huang, Kejing Du, Yajie Dai, Jia Wu, Linda Lim, Jason Goo, Yoshiki Ishida, Jiquan Liu, Jian Xu

**Affiliations:** ^1^ Procter & Gamble Singapore Innovation Center Singapore Singapore; ^2^ Procter & Gamble Beijing Innovation Center Beijing China; ^3^ Procter & Gamble Kobe Innovation Center Kobe Japan; ^4^ CAS Key Laboratory of Biofuels, Shandong Key Laboratory of Energy Genetics, Single‐Cell Center, Qingdao Institute of Bioenergy and Bioprocess Technology Chinese Academy of Sciences Qingdao Shandong China; ^5^ Shandong Energy Institute Qingdao Shandong China; ^6^ Qingdao New Energy Shandong Laboratory Qingdao Shandong China; ^7^ University of Chinese Academy of Sciences Beijing China

## Abstract

The impact of antibacterial detergent on microbial exchanges and its subsequent effect on malodor in used towels were examined. Homogenization of microbiome among postwashed and indoor dried towels that was dominated by known malodor‐producing bacteria. The microbial exchange was attenuated, and the abundance of malodor‐producing bacteria was reduced in towels laundered with antibacterial detergent. Reduction of malodorous volatile organic compounds produced from towels laundered with antibacterial detergent.

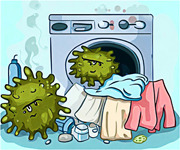

Apart from the removal of stains and soils, laundering also plays a critical role in the elimination and control of microbial contamination from garments, bedding linens, and cleaning towels. An effective antimicrobial intervention during the laundering process can influence the hygiene level of the washed textiles. In fact, numerous studies have demonstrated the survival and growth of microorganisms in various textile materials due to inadequate laundry hygiene [1, 2, 3, 4]. This can result in the generation of unpleasant odors via the metabolism of micro‐organisms that survived or regrew in the textiles after laundry. Indeed, microorganisms isolated from laundered dried clothes were reported to generate characteristic malodor volatile organic compounds (VOCs) consisting of branched unsaturated fatty acids and organosulfur compounds [5, 6, 7, 8, 9]. Although the presence of malodor‐generating micro‐organisms on textiles may originate from wear and usage, cross‐contamination from laundry processes can also contribute to the survival and buildup of these micro‐organisms even after laundry [10]. Conversely, the management of textiles—sorting of clothes before wash, laundered clothes, and their storage—may also have an impact on microbial cross‐contamination [10, 11].

The microflora and its composition on naturally soiled laundry have an important influence on the types and intensity of odorants generated by the odor‐generating microbiota [5, 12, 13]. The microflora of household laundry is diverse and unique as it is dynamically shaped by many factors including usage, textiles type, and laundry processes [10]. Understanding the impact of these factors on the laundry microflora is essential for defining guidelines for interventions to control malodor. Although several studies have shown that the diversity of textile microbiome is shaped by microbial exchanges during laundry in washing machines, the impact of microbial load after laundry has emerged as a significant constituent in malodor production [14–16], highlighting the importance of laundry regimes and detergent solution in microbial removal. Indeed, the reduction of microbes remains one of the most direct and widely used approaches for malodor reduction [17, 18]. However, the effect of antimicrobial detergent on the alteration of microbial community during laundry and its eventual malodor control has yet to be elucidated. Notably, previous understandings of microbial diversity in textiles have been limited to genus‐level identification, which is of insufficient resolution for pinpointing or distinguishing the underlying beneficial and harmful (i.e., pathogenic or malodor‐causing) microbial taxa [15, 16, 19]. Given that the degree of antimicrobial efficacy in textiles is highly dependent on the microbial species [20], knowledge of the types, composition, and abundance of malodor‐generating micro‐organisms at higher taxonomy specificity (e.g., the species‐level) is vital for evidence‐based microbial control and improvement of personal hygiene as related to textiles. Furthermore, the assessments of the antimicrobial efficacy of detergent on microbial contamination have been mostly restricted to lab‐scaled experiments where representative micro‐organisms are synthetically inoculated onto fabric swatches with artificial soil to simulate laundering conditions [1, 21, 22]. These studies have yet to elucidate the antimicrobial effect of detergents on malodor control in naturally soiled textiles.

To tackle these challenges, we examined the impact of antimicrobial detergents on microbial diversity and odorant production in naturally soiled cotton towels, by leveraging a novel metagenomic sequencing method that enables species‐level elucidation of low‐biomass microbial samples [23]. Collectively, we characterized the microflora from 124 cotton swatches treated by a standard mild detergent and antibacterial detergent at prewash, postwash and after‐indoor dry‐washed conditions across three independent wash batches (Figure 1). Additionally, we also measured microbial load and the abundance of the odorous dimethyl disulfide VOC from the towels. These efforts have provided mechanistic insights into bacteria‐caused malodor formation in washed clothes and underscored the importance of proper laundering for the control of malodor generation and proper personal hygiene.

**Figure 1:**
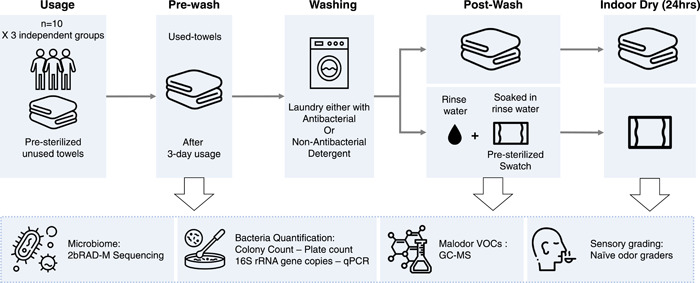
The schematic of the study design. Presterilized hand cotton towels were given to 30 individuals for 3‐day usage. The towels were randomly grouped into three wash batches (*n* = 10). Used towels were laundered either with a nonantibacterial or antibacterial detergent. The rinse water was collected where unused cotton swatches (*n* = 3) were soaked for 2 min with mixing. Samples were taken for analyses at prewash, postwash, and after indoor dry, as well as from rinse water from each treatment wash. Of note, the indoor dry condition was simulated in a conventional oven as described in Supporting Information. These samples were subjected to microbiome analysis, profiling of malodor volatile organic compounds (VOCs), bacteria quantification, and sensory grading evaluation. Illustrative icons used in the figures were from Flaticon.com.

## RESULTS

### Microbial transmission among towels during laundry resulted in microbiome homogenization

**Figure 2:**
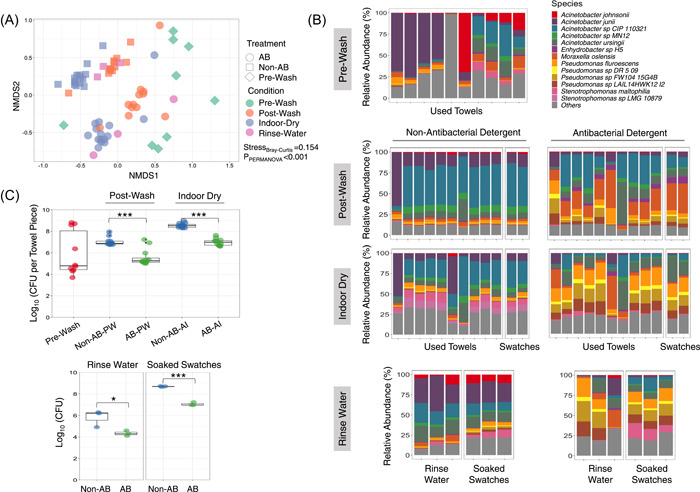
Microbial communities at the species level between treatment and condition groups based on wash batch 1. (A) Nonmetric multidimensional scaling plot based on Bray–Curtis distance. Permutational multivariate analysis of variance was used to measure the significance of difference for each condition. (B) Microbial composition of prewash used towels, postwash, after indoor dry and rinse water treated by the nonantibacterial detergent, and the antibacterial detergent was compared. Microbes with relative abundance <1% were grouped as “Others.” Each bar in the plots represents the microbial composition of towel used by the individuals at each washed condition. (C) Total bacterial load of towels was evaluated and compared by colony‐forming unit between the washes via the nonantibacterial and the antibacterial detergents at prewash, postwash, and after indoor dried washed conditions. Bacterial load of rinse water and pre‐sterilized swatches soaked in rinse water were also evaluated. AB, antibacterial; AI, after indoor dry; PW, postwash. Two sided Wilcoxon signed rank test: ***p < 0.001, *p < 0.1.

The microbiota of naturally soiled hand towels at various washed conditions—prewash, postwash, and after indoor dry—were examined at the species‐level using the 2bRAD‐M approach (Figure 1; Supporting Information; [23]). Intriguingly, the microbial communities among the prewash hand towels were highly dissimilar (Figure 2A,B), as reflected in the high beta diversity (Average [Avg] Bray–Curtis = 0.72) derived from pairwise microbiome comparison among the prewash towels. These observations are consistent across wash batches (Supporting Information: Figure S1A,B). Across the various washed conditions, bacteria were the prevailing micro‐organisms (>99%) detected on the cotton towels (Supporting Information: Table S1A). Notably, the microbiome of the prewash towels was dominated by *Acinetobacter* sp. (30.9%), *Pseudomonas* sp. (18.7%), and *Moraxella osloensis* (11.6%), which are common microbials found on textile materials (Supporting Information: Table S1B) [14, 16]. Human skin‐related microflora such as *Cutibacterium acnes* (2.1%), *Streptococcus* sp. (3.0%), and *Staphylococcus* sp. (0.9%) were also detected at a sizeable abundance, and are likely to originate from human skin through daily towel usage [24, 25].

Nonmetric multidimensional scaling analysis based on Bray–Curtis dissimilarity metric at the species level revealed distinctive shifts and segmentation of the microbial community in postwash as well as after indoor dry towels, which were further differentiated by the type of detergent used for laundry, *P*_PERMANOVA_ < 0.001 (Figure 2A; Supporting Information: Figure S1A). Interestingly, the microbial communities among after‐washed towels were extensively similar and homogenous (Figure 2B). This was consistent with repeated observations across independent wash batches (Supporting Information: Figure S1B), irrespective of the type of detergent used for laundry. The homogeneity of the microbial structure persisted after indoor drying albeit a shift in the communities when compared with after‐washed towels. Moreover, the presterilized cotton towel swatches that were laundered together with the used towels exhibited a similar microbial community after washing, further supporting the homogenization effect of the laundry. Interestingly, the microbiomes of postwash and indoor dried towels were dominated by known malodorous VOC‐producing bacteria such as *Moraxella osloensis* (18.1%), *Acinetobacter junii* (6.1%), *Acinetobacter ursingii* (5.6%), *Pseudomonas fluorescens* (4.9%), and *Stenotrophomonas rhizophila* (4.1%) [5, 26, 27, 28]. Collectively, these results suggest that the extensive microbial exchanges during laundry have resulted in the homogeneity of microbial communities among towels after washing, and after indoor drying.

To investigate the possible microbial exchanges from the laundry washing water to towels, we soaked presterilized cotton towel swatches into the rinse water collected during laundry for 2 min, and then recovered them after a 24‐h indoor drying period. The resulting microbial community on indoor dried cotton swatches was found to display high resemblance to that of the rinse water (Avg Bray–Curtis = 0.31) (Figure 2B), and correspondingly, preserved a moderate microbial community similarity with the after‐indoor‐dry used towels (Avg Bray–Curtis = 0.46). Together, these results holistically provide the evidence of microbial exchanges during laundry and suggest two possible modes of microbial transmission during the laundry process: (i) mechanical agitation via towel‐to‐towel contact and (ii) water‐to‐towel.

### Microbial transmission among towels was attenuated by the use of antibacterial detergent

To probe the effect of antibacterial detergent on microbial transmission during laundry, used towels together with pre‐sterilized cotton swatches were separately washed with two commercially available detergents: (i) a standard cleaning detergent with no antibacterial action, and (ii) detergent with antibacterial action during the washing process respectively. The microbial load of the swatches was measured through quantitative PCR (qPCR) of 16S ribosomal RNA (rRNA) gene, as well as culture‐based approaches. The distribution of microbial load of prewash towels was comparatively broader (standard deviation [SD] = 1.31) than the postwash towels, irrespective of detergent usage (SD = 0.42 for postwash towels with nonantibacterial; SD = 0.58 for those with antibacterial; Figure 2C; Supporting Information: Figure S2A,B). Interestingly, towels with low initial microbial loads before washing acquired more micro‐organisms after washing, while towels with high initial microbial loads showed a decrease after washing. This offset resulted in the swatches having a similar level of microbial load, conceivably due to the microbial exchanges during the laundry process, as reflected by the low microbial load SD and high microbial community similarity among the postwash towels.

The microbial load, measured by colony‐forming units (CFU), was reduced significantly, by 1.45 (log10 fold, *p* = 1.5 × 10^−7^), in those towels washed with the antibacterial detergent (Figure 2C). Such microbial load reduction was also validated with the qPCR of 16S rRNA gene copies (Supporting Information: Figure S2A). This observation was reproducible across wash batches. Microbial growth in the towels expectantly increased at relatively equal abundance, for both nonantibacterial (log10 fold CFU increase = 1.51) and antibacterial (log10 fold CFU increase = 1.41) detergent washes after 24 h of indoor drying. Furthermore, laundry with the antibacterial detergent appeared to reduce the microbial load (log10‐based CFU reduction by 1.47; *p* = 0.07) in the rinse water. The pre‐sterilized cotton swatches soaked in the rinse water from the antibacterial detergent also harbored lower amounts of microbial mass (log10‐based CFU reduction by 1.63; *p* = 1.7 × 10^−3^), indicating the reduced microbial transfer from rinse water to the soaked cotton swatches (Figure 2C).

To investigate the effect of usage of non‐antibacterial and antibacterial detergents on the microbial communities, we examined the beta‐diversity among the towels within each of the treatment groups. Microbiomes among towels washed with the antibacterial detergent displayed greater dissimilarity compared with those washed with the non‐antibacterial detergent. Indeed, the beta diversity among the antibacterial‐detergent washed towels (Bray–Curtis = 0.34) was significantly higher (*p* = 5.8 × 10^−05^) than the nonantibacterial‐detergent washed towels (Bray–Curtis = 0.18; Supporting Information: Figure S3A). Similarly, significant increments were also observed in the rinse water (*p* = 0.041) and soaked cotton swatches (*p* = 0.035) treated with the antibacterial detergent (Supporting Information: Figure S3A,B). In addition, towels washed with the antibacterial detergent exhibited a more diverse (as revealed by the Richness index and the Shannon index) microbial community (Supporting Information: Figure S3C). This observation was also consistent with the results from wash batch 2 (Supporting Information: Figure S3C). These results suggest that the use of antibacterial detergent could have moderated the microbial transmission during laundry process by reducing the microbial load in both towels and rinse water. This allows the towels to retain its prewash microbiome, and thus greater microbiome dissimilarity was observed among the towels washed with antibacterial as compared with those washed by the nonantibacterial detergent.

### Antibacterial detergent reduced malodor‐generating organisms and led to noticeable sensory effects

**Figure 3:**
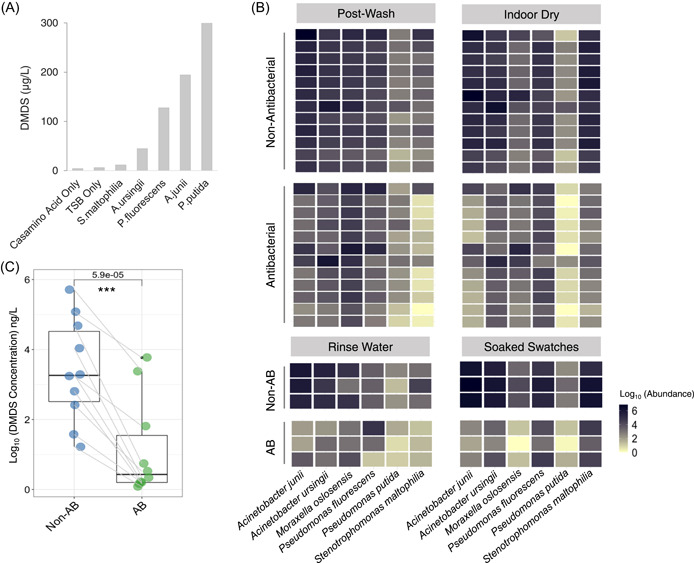
Malodor‐generating bacterial species detected and isolated from the used and washed towels. (A) Dimethyl disulfide (DMDS) production from headspace from the cultures of bacterial species isolated from used towels. (B) Heatmaps of the estimated abundance of malodor‐generating bacteria in postwash towels, indoor dried towels, rinse water, and soaked swatches. (C) DMDS evaluation on headspace of swatches washed with the nonantibacterial and the antibacterial detergents. The evaluation was based on randomly selected used towels (*n* = 10), as assessed by GC‐MS. AB, antibacterial; GC‐MS, gas chromatography‐mass spectrometry; TSB, tryptic soy broth. Two sided Wilcoxon signed rank test: ***p < 0.001.

Elucidation of microbial composition at the species level can improve the specificity in identifying malodor‐causing microbes on consumer‐used towels. In our study, we detected the presence of *Acinetobacter junii* (10.8%), *Acinetobacter ursingii* (2.7%), *Moraxella osloensis* (11.5%), *Stenotrophomonas maltophilia* (2.9%), *Pseudomonas fluorescens* (2.8%), and *Pseudomonas putida* (1.0%) in prewash used towels at sizeable abundance across all batches (Supporting Information: Table S1). These bacteria are known to produce an assortment of foul‐smelling VOCs such as dimethyl disulfide, dimethyl trisulfide, 3‐methylbutanoic acid and 4‐methyl‐3‐hexenoic acid [5, 27, 28], which have been detected from soiled clothing [9]. Except for *Moraxella osloensis*, we were able to isolate the five malodor‐generating bacteria identified from the metagenomics analysis of the prewash towels and cultured them independently in growth media for 48 h. We then measured the headspace from the bacterial cultures and detected the production of dimethyl disulfide (range 10–300 ng/L) (Figure 3A). This finding supports the notion that these bacteria can produce malodorous VOCs on towels under suitable conditions, with dimethyl disulfide being a significant contributor to laundry indoor‐drying malodor based on headspace analysis of indoor‐dried consumer laundry.

Furthermore, to assess the impact of antibacterial detergent on malodor production in towels, we examined the abundance of known malodor‐generating bacteria detected in prewash, postwash and after indoor dry towels. The abundance of these bacteria was significantly lower in towels washed with the antibacterial detergent compared to those washed with non‐antibacterial detergents (Figure 3B). This reduction was observed consistently across all wash batches (Supporting Information: Figure S4A), in towels after indoor drying, rinse water and cotton swatches washed with the antibacterial detergent (Figure 3B). The measurement of headspace concentration of dimethyl disulfide from postwash towels showed that towels washed with the antibacterial detergent have produced less dimethyl disulfide than those towels washed with the non‐antibacterial detergent (Figure 3C). In addition, five sensory panelists were engaged to evaluate the odor perception of each corresponding towels (5 × used towels, 2 × washed pre‐sterilized cotton swatches, and 3 × rinse water‐soaked cotton swatches) that were washed with the nonantibacterial and the antibacterial detergents. It was revealed that after‐indoor‐dried cotton swatches washed with the antibacterial detergent were perceived to be more sensorially pleasing than those washed with nonantibacterial detergent (Supporting Information: Figure S4B). Taken together, these results underscore the profound effect of reducing malodor‐producing bacteria during laundry on VOCs production in postwash fabric, and importantly, the translation of such bacterial reduction into noticeable perceived pleasant sensory effect for consumers.

## DISCUSSION

In this study, we explored the species‐resolution microbiome profiles of textiles during the laundry process. Through the assessment of microbiome profiles in naturally soiled cotton swatches at three laundry wash conditions—prewash, postwash, and after indoor dry, we have uncovered microbial admixture among the towels during laundry that mirrors the findings of a previous study [14]. Consistent across the three independent batches, the microbial exchanges during laundry had resulted in high microbiome homogeneity among postwash and after indoor dry swatches, where drastic shifts in the microbial community were observed as compared with the corresponding prewash swatches. Interestingly the microbial diversity and composition in postwashed towels appeared to vary in a stochastic manner across the three batches. This phenomenon may possibly be explained by the stochastic nature in the placement of towels in the washing machine and the subsequent contacts during the laundry process.

Using the 2bRAD‐M metagenome sequencing approach, we had resolved the microbiome of cotton swatches at the species‐level. This has enabled the unbiased detection of six known malodor‐generating bacterial species—*Acinetobacter junii*, *Acinetobacter ursingii*, *Moraxella osloensis*, *Stenotrophomonas maltophilia*, *Pseudomonas fluorescens*, *Pseudomonas putida*—in the towels. Furthermore, five of these bacterial species were isolated from the used cotton swatches and were validated to produce the malodorous dimethyl disulfide VOC after the culturing in growth media. The formation of dimethyl disulfide can occur through microbial‐mediated degradation of sulfur‐containing amino acids (methionine and cysteine) to emit methanethiol which undergoes subsequent rapid autoxidation to form dimethyl disulfide or dimethyl trisulfide independent of microbial actions [29, 30]. Beside dimethyl disulfide, numerous VOCs such as butyric acid, 3‐meythylbutanoic acid, 4‐methyl‐3‐hexenoic acid and hexanal were also detected on textiles [5, 7, 31], implying that the overall malodor intensity and types were likely contributed by the collective actions of microbial community on the textiles. Meanwhile, it is important to note that the metagenome‐sequencing approach employed for microbiome dissection here is inherently limited as it does not distinguish between live and dead of cells. Therefore, one future direction would be to employ single‐cell metabolic phenome approaches such as the Ramanome technology platforms [32, 33, 34] for (i) profiling the in situ microbial metabolic activities such as vitality, substrate intake rate, product profile and response to stress [32], (ii) establishing the link between in situ metabolic phenome and genome at precisely one‐cell resolution via RACS‐Seq [35, 36], and (iii) isolating the individual cells with targeted metabolic phenome via RACS‐Culture [37], which is a “screen first and culture second” strategy for efficiently mining functional microbes from environmental samples.

An important aspect of laundering is to control malodor through the reduction of malodor‐generating micro‐organisms and their metabolites on textiles. Generally, antibacterial efficacy is delivered via the action of anionic surfactant that functions as bacterial membrane disruptor [38, 39]. However, at the concentration used in laundry, its bactericidal efficacy is limited due to the lack of electrostatic affinity with the bacterial cells [40]. The antibacterial detergent used in our study overcomes this by the inclusion of pH adjuster in the form of citric acid which will alter the bacterial cell surface charge from negative to neutral or positive, and hence enables the interaction of anionic surfactant with the bacterial cells [40]. In addition, the low pH condition itself can further enhance the detergent antibacterial activity [41]. Indeed, a difference in the pH of the rinse water laundered by nonantibacterial (pH = 6.16) or antibacterial (pH = 4.46) detergent was noted.

## CONCLUSION

Collectively, the antibacterial detergent impacts the microbial exchanges via pH‐dependent antibacterial activity in rinse water and towels during laundry, and significantly reduces micro‐organisms after wash, particularly the malodor‐generating bacteria. Importantly, such reduction in the malodor‐generating bacteria can be translated into better sensory outcome for humans. Overall, our study has holistically decoded the mechanism on how antibacterial detergents can deliver deodorization benefits for consumer‐used towels by reducing VOCs production and improving the overall sensory outcome.

## METHODS

### Experimental design, execution & sampling

Pre‐sterilized new hand towels (28 × 28 cm) were given to 30 individuals (Osaka, Japan), and collected back after 3‐day usage. The hand towels were sterilized using autoclave machine (Tuttnauer 3870ELV) at 121°C for 15 min. Each used towel was then cut into two pieces (14 × 28 cm) where were respectively subjected to wash with nonantibacterial detergent (Polyoxyethylene alkylether, Linear alkyl benzene sulphonate, Fatty acid, Stabilizer, Alkalifying agent, Dispersing agent, Water softener, Brightener, Enzyme, product pH value 8.7) or antibacterial detergent (Polyoxyethylene alkylether, Linear alkyl benzene sulphonate, pH adjuster/Citric acid, Stabilizer, Dispersing agent, Alkalifying agent, Brightener, product pH value 2.5). The study was conducted with the towels processed and handled across three batches. The washing was conducted using a new commercial small‐scale washing machine (AUX, HB20P26‐31) at the following conditions—1000 ppm detergent dosage (1 g product/L water) as per product label recommendation, main wash 10 min, rinse 3 min × two cycles, each treatment group was washed independently in 6 L of purified water (milli‐Q IQ 7000; MERCK) at 25°C. The bacterial loads and microbiome profiles of the towels were examined at two different touchpoints: prewash and postwash. Before each wash, the washing machine was disassembled and sterilized with 75% ethanol, therefore microbial transfer from washing machine was minimal.

Moreover, presterilized cotton swatches (*n* = 2, 14 × 14 cm) were also added into each wash. The washed towels were subjected to incubation (MIR‐554 Cooled Incubator; SANYO) for 24 h at 25°C with the relative humidity maintained at >90% which simulates indoor drying (Figure 1). In addition, the 1st rinse water from each treatment wash was collected where presterilized cotton swatches (*n* = 3, 14 × 14 cm) were soaked for 2 min with mixing. The bacterial loads and microbiome profiles of 1st rinse water and the soaked towels (after 48‐h incubation) were examined. In total, 124 samples were collected (Supporting Information: Table S1) and examined for the bacterial loads and microbiome profiles. All samples were sealed and stored in Ziploc bags at −80°C after collection before downstream processing.

More details are provided in Supporting Information.

## AUTHOR CONTRIBUTIONS

TzeHau Lam, Yuxiang Liu, Jason Goo, Yoshiki Ishida, Fumi Iuchi, Yolanda Huang, Jiquan Liu, and Jian Xu conceived and devised the overall research goals and aims. TzeHau Lam, Yuxiang, Liu, Fumi Iuchi, and Yolanda Huang planned and executed the study implementation. Yuxiang Liu, Jia Wu, Yajie Dai, and Kejing Du contributed to the sampling processing and performed the laboratory experiments. TzeHau Lam and Yuxiang Liu processed the experimental data, performed the analysis and interpretation of the results. TzeHau Lam, Jiquan Liu, and Jian Xu wrote the manuscript in consultation with Linda Lim, Jason Goo, Yoshiki Ishida, Yolanda Huang, Kejing Du, and Yajie Dai. Linda Lim, Jason Goo, Jiquan Liu, TzeHau Lam, and Jian Xu provided the overall project supervision. All authors read and approved the manuscript.

## CONFLICT OF INTEREST STATEMENT

TzeHau Lam, Yuxiang Liu, Fumi Iuchi, Yolanda Huang, Kejing Du, Jia Wu, Linda Lim, Jason Goo, Yoshiki Ishida, and Jiquan Liu are employed by Procter & Gamble.

## Supporting information

Supporting information.

## Data Availability

All sequencing data generated in this study are available on PRJEB58810 (https://www.ebi.ac.uk/ena/browser/view/PRJEB58810). Supporting Information (figures, tables, scripts, graphical abstract, slides, videos, Chinese translated version, and updated materials) may be found in the online DOI or iMeta Science http://www.imeta.science/. All sequencing data generated in this study are available on PRJEB58810.
